# Tumbly: An Open-Source Home-Cage Device for Time-Restricted Feeding in Mice

**DOI:** 10.64898/2026.10.19.760239

**Published:** 2026-10-24

**Authors:** Mason R. Barrett, Cynthia Chen, Winston Li, Matt Gaidica, Amy K. Wagner, T. John Wu, Erik S. Musiek, Alexxai V. Kravitz

## Abstract

Time-restricted feeding (TRF) is a common tool for studying metabolism, circadian rhythms, aging, and neurodegeneration. Here, we describe a new device called Tumbly, an automated device for performing TRF studies in mouse home-cages. Tumbly controls access to rodent diets at user-set times via a programmable servo-driven food hopper. Tumbly is also battery powered and compatible with multiple types of vivarium home-cages, due to a removable faceplate that can be customized to fit different cage dimensions without modification. Finally, Tumbly is open-source and affordable, with all design files available online, allowing researchers to build and customize it. As a validation test, we deployed Tumbly in two labs at separate institutions, one with singly housed mice (n = 6, within-subject) and one with group-housed mice (n = 22, between-group). We measured daily body weight and food intake. In both labs, mice on an 8-hour TRF schedule maintained their body weight and ate similar amounts of food compared to ad libitum feeding. Our quantified error rates were also low, with no device failures over >1000 hours of operation. We conclude that Tumbly provides a reliable, low-cost option for running TRF studies in mice.

**Significance Statement:**

Time-restricted feeding (TRF) studies are often run by hand, moving food or animals between cages at fixed times to restrict feeding. This approach limits the study cohort size to what experimenters can manage, requires someone to be present to control food at inconvenient hours, and can add stress to the animals from repeated experimenter interventions. Tumbly removes these limitations by automating the TRF procedure. Tumbly is also open-source and can be modified to adapt it to more complex monitoring experiments, such as adding in-cage temperature or humidity sensors. Finally, because Tumbly is affordable, it makes automation of TRF both more accessible to labs with smaller budgets and scalable for researchers that need to run high throughput TRF studies.

